# Clinical Significance of the Prognostic Nutritional Index for Predicting Short- and Long-Term Surgical Outcomes After Gastrectomy: A Retrospective Analysis of 7781 Gastric Cancer Patients

**DOI:** 10.1097/MD.0000000000003539

**Published:** 2016-05-06

**Authors:** Jee Youn Lee, Hyoung-Il Kim, You-Na Kim, Jung Hwa Hong, Saeed Alshomimi, Ji Yeong An, Jae-Ho Cheong, Woo Jin Hyung, Sung Hoon Noh, Choong-Bai Kim

**Affiliations:** From the Department of Surgery (JYL, HIK, YNK, JYA, JHC, WJH, SHN, CBK), Yonsei University College of Medicine; Open NBI Convergence Technology Research Laboratory (HIK), Severance Hospital; Biostatistics Collaboration Unit (JHH), Yonsei University College of Medicine, Yonsei University Health System, Seoul, Korea; Department of Surgery (SA), King Fahd Hospital of the University Khobar, Khobar, Saudi Arabia; Brain Korea 21 Project for Medical Science (WJH, SHN), Yonsei University College of Medicine; and Robot and Minimally Invasive Surgery Center (WJH), Yonsei University Health System, Seoul, Korea.

## Abstract

To evaluate the predictive and prognostic significance of the prognostic nutritional index (PNI) in a large cohort of gastric cancer patients who underwent gastrectomy.

Assessing a patient's immune and nutritional status, PNI has been reported as a predictive marker for surgical outcomes in various types of cancer.

We retrospectively reviewed data from a prospectively maintained database of 7781 gastric cancer patients who underwent gastrectomy from January 2001 to December 2010 at a single center. From this data, we analyzed clinicopathologic characteristics, PNI, and short- and long-term surgical outcomes for each patient. We used the PNI value for the 10th percentile (46.70) of the study cohort as a cut-off for dividing patients into low and high PNI groups.

Regarding short-term outcomes, multivariate analysis showed a low PNI (odds ratio [OR] = 1.505, 95% CI = 1.212–1.869, *P* <0.001), old age, male sex, high body mass index, medical comorbidity, total gastrectomy, and combined resection to be independent predictors of postoperative complications. Among these, only low PNI (OR = 4.279, 95% CI = 1.760–10.404, *P* = 0.001) and medical comorbidity were independent predictors of postoperative mortality. For long-term outcomes, low PNI was a poor prognostic factor for overall survival, but not recurrence (overall survival: hazard ratio [HR] = 1.383, 95% CI = 1.221–1.568, *P* < 0.001; recurrence-free survival: HR = 1.142, 95% CI = 0.985–1.325, *P* = 0.078).

PNI can be used to predict patients at increased risk of postoperative morbidity and mortality. Although PNI was an independent prognostic factor for overall survival, the index was not associated with cancer recurrence.

## INTRODUCTION

Successful treatment of gastric cancer largely depends on a successful gastrectomy. While this surgery can potentially cure the disease, it also harbors the risk of perioperative morbidity and mortality. Perioperative complication rates during gastric cancer surgery range from 10% to 46%,^1–4^ and adversely affect long-term survival.^5,6^ As gastric cancer is the fifth most common malignancy worldwide,^7^ improving short- and long-term surgical outcomes for patients with gastric cancer is of great necessity.

Researchers have spent great effort to identify factors related to adverse surgical outcomes and prognosis. Several factors, including medical comorbidity, old age, combined resection, and advanced stage, are associated with surgical outcomes and hold prognostic significance^8–10^; however, these factors are primarily unamenable, as they are related to the patient's physical or disease status. Thus, assessments of nutritional status have emerged as potential prognostic factors, since nutritional status can be corrected prior to surgery. While several tools for assessing nutritional status have been evaluated, including the nutritional risk index,^11^ the nutritional risk screening 2002,^12^ and subjective global assessment,^13,14^ these are difficult to use in daily clinical practice due to their complexity. Moreover, some of the parameters used by these tools are not always available, for example changes in weight.

Unlike other assessments, the prognostic nutritional index (PNI) can be easily calculated using the following equation: [(10 × serum albumin (g/dL)) + (0.005 × total lymphocyte count)].^15^ The parameters used by this index are routinely evaluated in laboratory tests during preoperative diagnostic workup and are easy to repeat. The predictive value of the PNI for surgical outcomes is widely accepted in various solid organ cancers, including esophageal, colorectal, liver, and pancreatic cancer.^16–19^ However, only a few reports have evaluated the significance of PNI in predicting short- and long-term surgical outcomes for patients with gastric cancer,^20,21^ and a comprehensive study has never been conducted. Furthermore, controversy exists regarding the optimal cut-off values for PNI in predicting short- and long-term surgical outcomes.

Accordingly, the aim of this study was to assess the value of PNI as a predictor of perioperative morbidity and mortality, as well as a prognostic factor for recurrence-free and overall survival. Additionally, we also compared the performance of a single cut-off value based on percentiles of PNI to statistically optimized cut-off values of PNI for individually predicting morbidity, mortality, recurrence-free survival, and overall survival.

## METHODS

### Patients

In the present study, we retrospectively reviewed and analyzed data from medical records stored in a prospectively maintained database. Our study included 8811 consecutive patients with histologically confirmed gastric adenocarcinoma who underwent gastrectomy at Severance Hospital between January 2001 and December 2010. We excluded 1030 patients with a history of other primary cancer, neoadjuvant chemotherapy, radiotherapy, noncurative resection, or emergency surgery due to perforation, bleeding, or obstruction. The remaining 7781 patients were included for analysis. The Institutional Review Board of Severance Hospital approved this study and waived the need for written informed consent from the participants (4-2015-0085).

Clinicopathological characteristics included age, sex, preoperative body mass index (BMI), medical comorbidities, American Society of Anesthesiologists (ASA) score, tumor size, and pathological stage. A medical comorbidity was defined as a preexisting medical condition that needed long-term treatment. Perioperative data were also noted, including the extent of resection, combined resection, and operation time. Surgical resection and extent of lymphadenectomy were performed in accordance with the Japanese guidelines for treating gastric cancer.^22^ Adverse events occurring within 30 days after surgery or during hospitalization were classified as postoperative complications or mortality; the type of complication was recorded. Patient staging was adjusted according to the 7th edition of the American Joint Committee on Cancer staging system.^23^ Follow-up evaluations were performed according to a fixed schedule: every 3 months for 2 years, and then every 6 months for 3 years thereafter. Follow-up evaluations comprised clinical and laboratory examinations with biannual imaging and annual endoscopic evaluation. Patients with stage II or higher disease were recommended to receive 5-fluorouracil-based adjuvant chemotherapy.

### Prognostic Nutritional Index and Patient Grouping

We obtained laboratory data, including serum albumin levels and lymphocyte counts, from baseline workup conducted within 2 months before surgery. The PNI was calculated by the following equation: [(10 × serum albumin (g/dL)) + (0.005 × total lymphocyte count)]. First, we divided patients according to every 5th percentile of PNI into 20 groups (389 patients in each group). From the 5th to 100th percentiles, the mortality events in each group were: 3, 5, 2, 1, 0, 1, 3, 0, 0, 1, 0, 1, 0, 0, 1, 0, 2, 0, 1, and 1, respectively. The complication rates for each of these groups were: 18.0%, 17.2%, 11.1%, 10.2%, 12.3%, 12.8%, 9.8%, 12.3%, 10.0%, 7.2%, 11.1%, 9.5%, 9.9%, 9.4%, 10.7%, 13.3%, 11.2%, 10.8%, 9.3%, and 11.90%, respectively. As the 10th percentile of PNI showed the highest morbidity and mortality, we used its value as a cut-off to divide patients into 2 groups: higher or lower than the PNI value for the 10th percentile. We hypothesized that this value would be more practical than median or mean values and could better identify patients at high risk for perioperative morbidity, as well as those who may benefit from nutritional interventions prior to surgery.

### Statistical Analysis

Categorical variables were compared using the *χ*^2^ test, and continuous variables were compared using Student *t* test. We used Youden indices to determine the optimal PNI cut-off values to maximize sensitivity and specificity for complications and mortality.^24^ Comparison of the area under ROC the curve (AUC) was performed as recommended by DeLong et al.^25^

Overall survival was defined as the duration of time from the date of surgery until the date of patient death. Recurrence-free survival was defined as the duration of time from the date of surgery until the date of histologic or radiologic recurrence of gastric cancer. To find the optimal cut-off PNI values for overall and recurrence-free survival, we used the Contal and O’Quigley method, which is based on the concept of maximizing the log-rank statistic.^26^ We then compared the integrated areas under the curve (iAUC) between the model divided according to the 10th percentile and the models divided according to the optimized cut-off values determined using the Contal and O’Quigley method. iAUC is a weighted average of the AUC across a follow-up period and is a measure of the predictive accuracy of a model during follow-up. A higher iAUC indicates a better predictive accuracy. Differences in iAUC were calculated using a bootstrapping method with 1000 resampling times.^27^

All *P* values less than 0.05 were regarded as significant, and all statistical tests were 2-sided. Analyses were conducted using SAS software (version 9.2; SAS Institute, Cary, NC) and R software (version 2.13.1; R Foundation for Statistical Computing, Vienna, Austria).

## RESUTLS

### Patient Demographics and Comparison of the Low and High PNI Groups

Table 1 lists the clinical, laboratory, operative, and pathologic characteristics of the entire cohort and compares the characteristics for the low versus the high PNI group. Among the entire cohort, 3624 were older than 60 years (46.6%); 5150 were male (66.2%); the mean BMI was 23.2 ± 3; and 3366 had a medical comorbidity (43.3%). Subtotal and total gastrectomies were performed in 5895 (75.8%) and 1886 (24.2%) patients, respectively. Combined resection was performed in 280 patients (3.6%). Stage I, II, and III disease was found in 4608 (59.2%), 1286 (16.5%), and 1887 (24.3%) patients, respectively. The mean PNI was 54.2 ± 5.9.

**TABLE 1:**
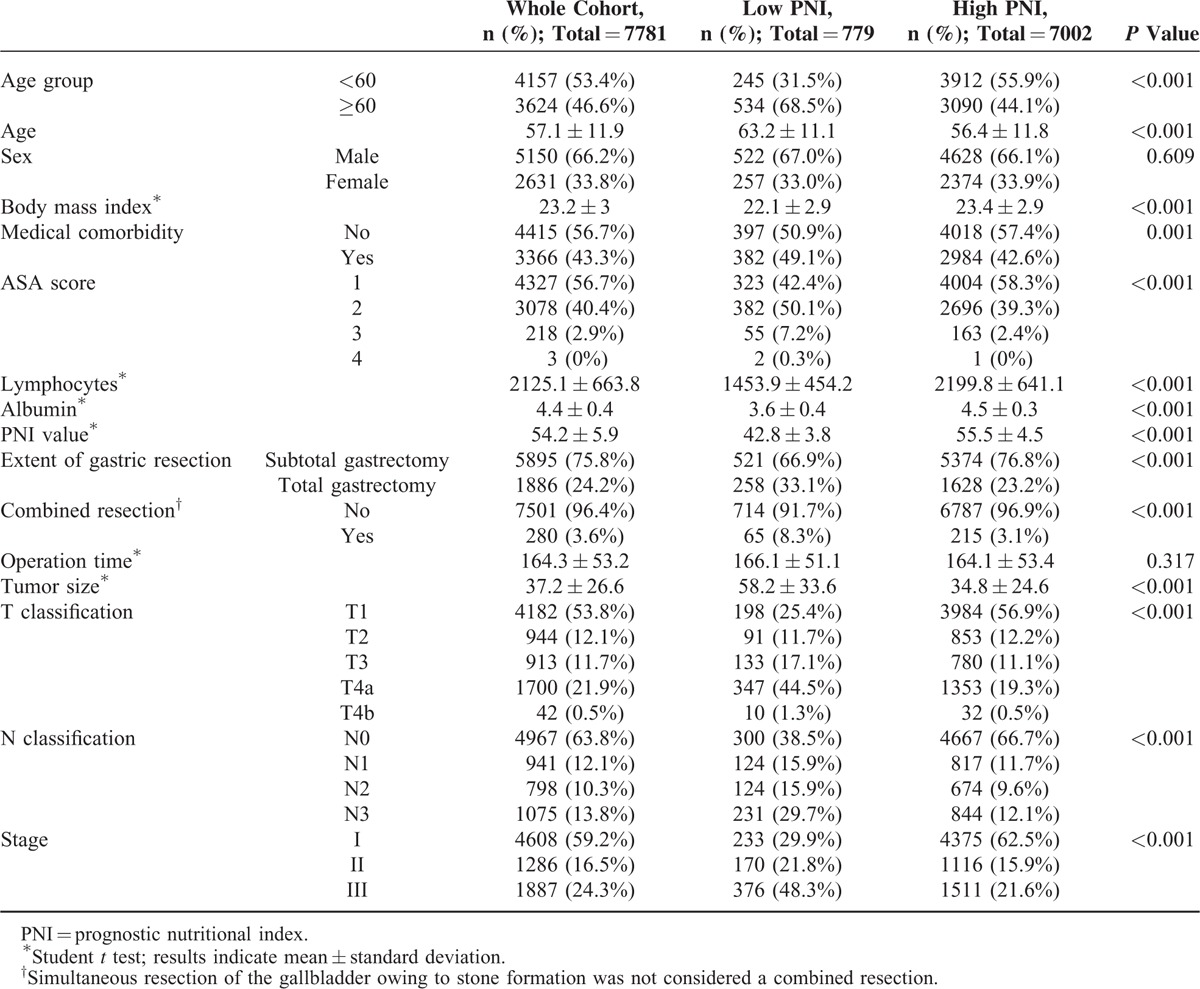
Demographics of Patients According to PNI Group

Grouping patients according to the PNI value of 46.70, we found that low PNI was associated with old age, low BMI, medical comorbidity, a higher ASA score, low lymphocyte counts, and low albumin levels. The mean age of the patients with low and high PNI was 63.2 ± 11.1 and 56.4 ± 11.8, respectively. Operative parameters showed more frequent association between patients with a low PNI and total gastrectomy or combined resection than those with a high PNI. Patients with low PNI also had larger tumors, more advanced T and N classifications, and more advanced disease stage.

### Comparison Between the 10th Percentile and Statistically Optimized Cut-Off Values of PNI

Using AUC values, we compared the performance of the 10th percentile PNI value versus statistically optimized PNI cut-off values to assess overall complications, mortality, recurrence-free survival, and overall survival (Table 2). For short-term surgical outcomes, the optimal cut-off values determined using Youden's method for morbidity (PNI = 51.52) and mortality (PNI = 52.18) had higher AUCs than that of the 10th percentile value (PNI = 46.70). However, no statistical difference was observed for the prediction of an event. Regarding long-term surgical outcomes, the optimal cut-offs determined by the Contal and O’Quigley method for recurrence-free survival (PNI = 53.22) and overall survival (PNI = 52.36) had higher iAUCs with statistically better predictive power (recurrence-free survival: ΔAUC = 0.034, 95% CI = 0.021–0.046; overall survival: ΔAUC = 0.029, 95% CI = 0.014–0.042) than that of the 10th percentile value.

**TABLE 2:**
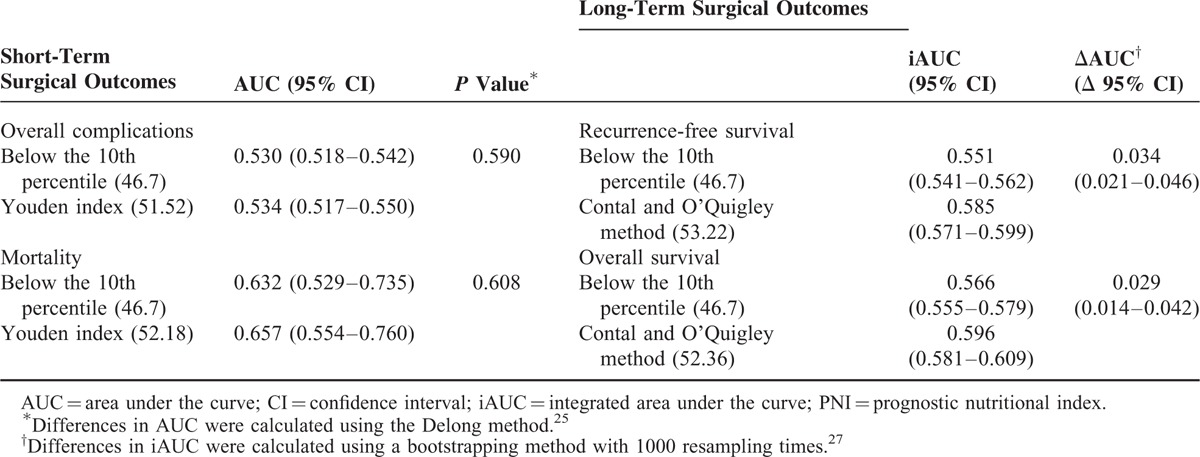
Performance of the 10th Percentile Value in Comparison With Statistically Optimized Cut-Off Values of PNI

### Short-Term Surgical Outcomes

Patients in the low PNI group remained in the hospital longer than those in the high PNI group (Table 3). The overall complication and mortality rates for the entire cohort were 11.4% and 0.3%, respectively. Compared with the high PNI group, the low PNI group showed significantly higher complication rates (10.7% versus 17.6%, respectively; *P* < 0.001) and mortality rates (0.2% versus 1%, respectively; *P* < 0.001). The low PNI group had higher rates of wound infection, abscess formation, intraluminal bleeding, intestinal obstruction, and leakage than the high PNI group. Complications associated with pulmonary, renal, hepatic, and cardiac organs also were observed frequently in the low PNI group. Logistic regression analysis revealed that low PNI (odds ratio [OR] = 1.505, 95% CI = 1.212–1.869, *P* < 0.001), old age, male sex, high BMI, medical comorbidity, total gastrectomy, and combined resection were independent risk factors for overall complications (Table 4). Only low PNI (OR = 4.279, 95% CI = 1.760–10.404, *P* = 0.001) and medical comorbidity were independent risk factors for mortality.

**TABLE 3:**
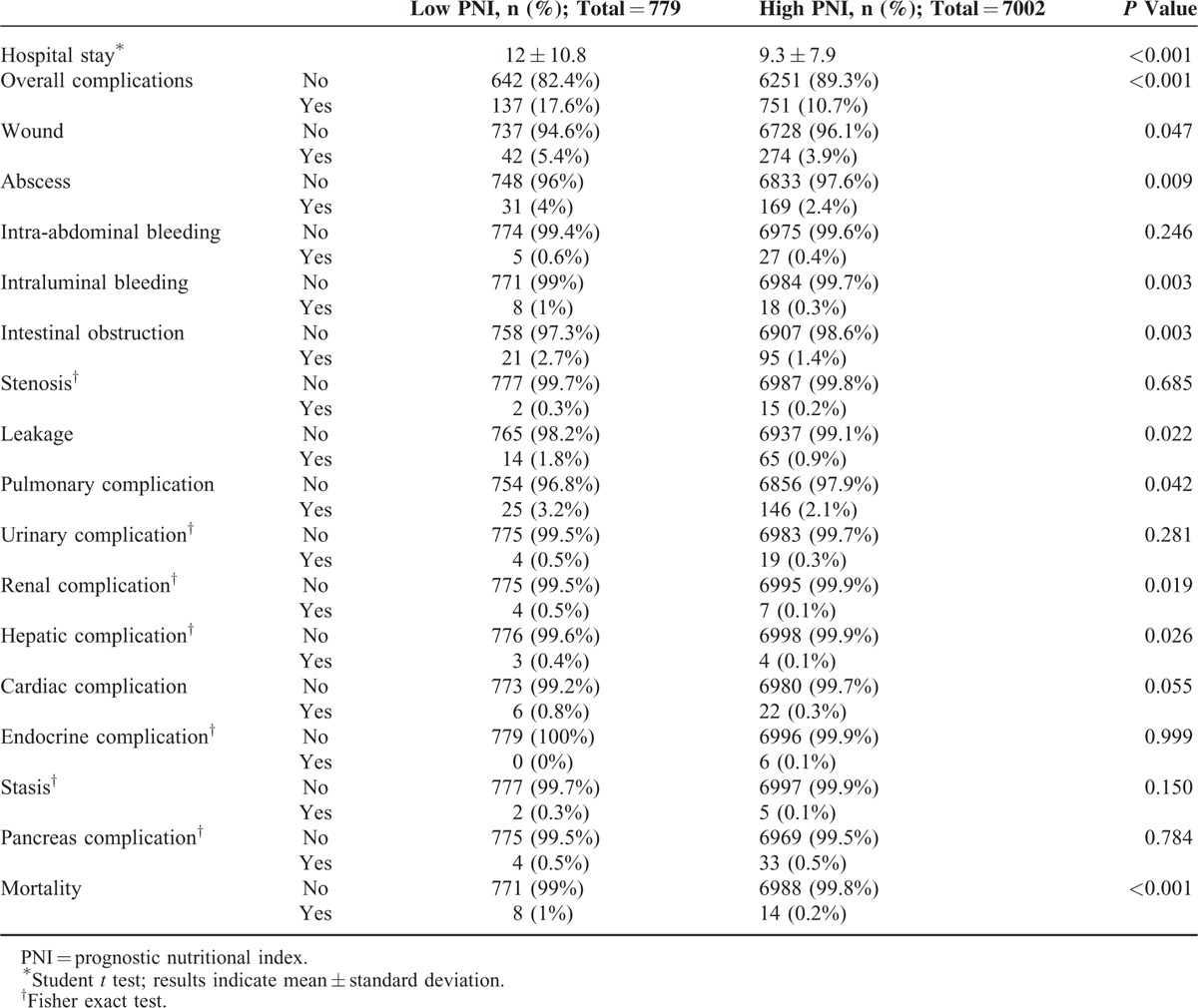
Short-Term Surgical Outcomes According to PNI Group

**TABLE 4:**
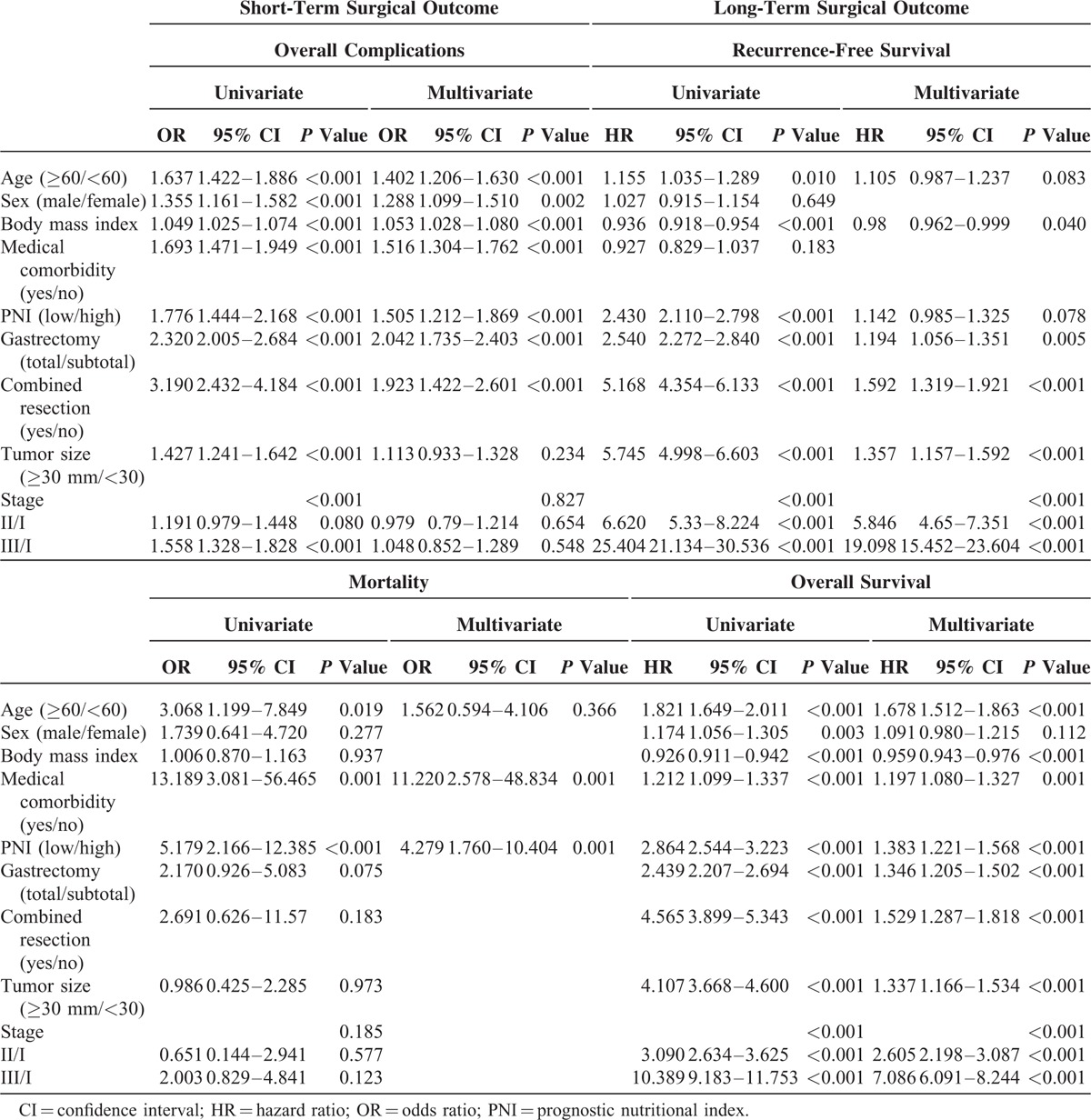
Univariate and Multivariate Analyses of Short- and Long-Term Surgical Outcomes

### Long-Term Surgical Outcomes

**FIGURE 1:**
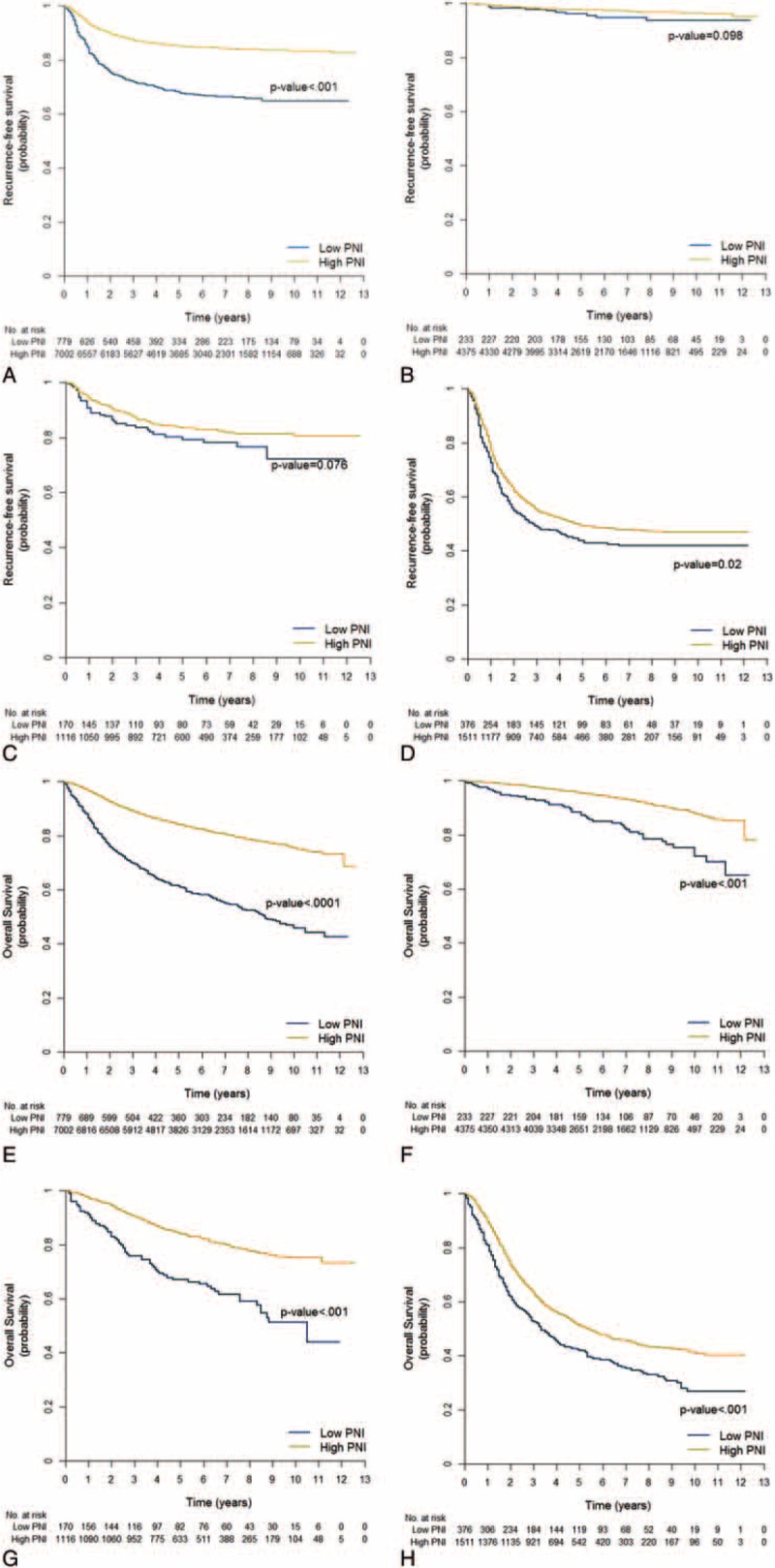
Long-term surgical outcomes according to PNI group at each stage of disease. Recurrence-free survival of (A) all stages, (B) stage I, (C) stage II, and (D) stage III. Overall survival of (E) all stages, (F) stage I, (G) stage II, and (H) stage III. HR = hazard ratio; PNI = prognostic nutritional index.

In the recurrence-free survival analysis, patients in the low PNI group had a poor prognosis (Figure 1A; *P* < 0.001). However, after stratifying patients according to disease stage, we found no significant differences between the low and high PNI groups in recurrence-free survival for patients with stage I or stage II disease (Figure 1B–D; stage I, *P* = 0.098; II, *P* = 0.076; III, *P* = 0.020). Further stratifying stage III into stages IIIa, IIIb, and IIIc also revealed no significant survival differences between the low and high PNI groups (*P* = 0.606, *P* = 0.461, and *P* = 0.533, respectively). Applying the optimal PNI value determined by the Contal and O’Quigley method, recurrence-free survival still showed no survival difference between low and high PNI groups stratified by disease stage, with the exception of stage Ia (Ia, *P* = 0.008; Ib, *P* = 0.641; IIa, *P* = 0.251; IIb, *P* = 0.116; IIIa, *P* = 0.536; IIIb, *P* = 0.099; and IIIc, *P* = 0.677). Regardless of the cut-off value applied, PNI was not associated with recurrence-free survival. Using Cox regression analysis, we found that low BMI, total gastrectomy, combined resection, larger tumor size, and stage of disease were independent risk factors of recurrence-free survival (Table 4). Low PNI was not an independent risk factor for recurrence-free survival (hazard ratio [HR] = 1.142, 95% CI = 0.985–1.325, *P* = 0.078).

In the overall survival analysis, the low PNI group had a poor prognosis for all stages of disease (Figure 1E–H; for all stages and stages I, II, and III: *P* < 0.001). Independent risk factors for overall survival included low PNI (HR = 1.383, 95% CI = 1.221–1.568, *P* < 0.001), old age, low BMI, medical comorbidity, total gastrectomy, combined resection, larger tumor size, and disease stage.

### Comparison After Adjustment for Confounding Factors

To account for confounding factors in evaluating the performance of each cut-off value, we applied a stepwise adjustment for confounding factors to develop models for short- and long-term surgical outcomes (Table 5). Both the 10th percentile cut-off value and the statistically optimized cut-off values showed robustness after adjusting for confounding variables. Interestingly, the 10th percentile value showed higher odds ratios and hazard ratios with more statistical significance than the statistically optimized cut-off values for mortality, recurrence-free survival, and overall survival.

**TABLE 5:**
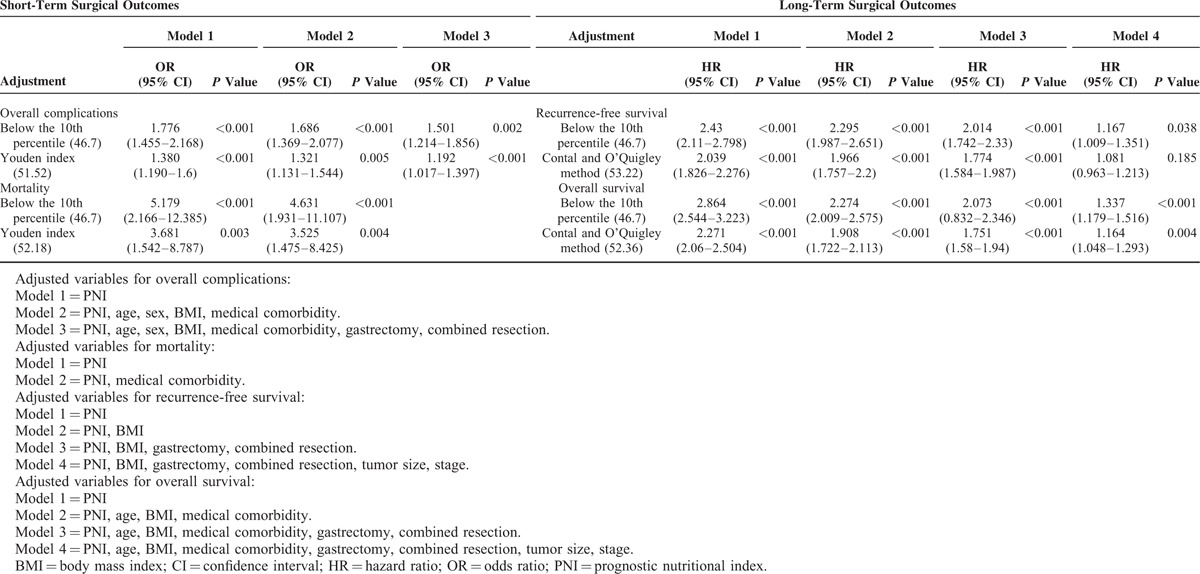
Performance of the Cut-Off Values After Adjustment for Confounding Factors

## DISCUSSION

The present study retrospectively analyzed individual clinical data from 7781 patients who underwent curative resection for gastric cancer at a high-volume center in Korea. Selecting the 10th percentile PNI value as a cut-off, we found that patients with a PNI lower than 46.70 show significantly higher overall morbidity and mortality than those with a higher PNI. Low PNI also was associated with unfavorable overall survival; recurrence-free survival was not correlated with PNI.

In the literature, various cut-off values for PNI have been suggested, including 49.7,^28^ 48,^21^ and 44.7.^20^ In our study, we used the 10th percentile PNI value (46.70) and statistically optimized values as cut-offs for overall complications (51.52, the 28.4th percentile), mortality (52.18, the 32.3rd percentile), recurrence-free survival (53.22, the 39.6th percentile), and overall survival (52.36, the 33.4th percentile). Clinically, a cut-off value higher than the 10th percentile value, including median or mean values, would not be useful, as too many patients would be categorized as high risk. In this study, patients with a PNI value in the 10th percentile showed a mortality rate 5 times higher than that of other patients. As strength of our study, we were able to validate the use of PNI to predict mortality, which is an extremely rare event, in a very large cohort. Additionally, we successfully demonstrated the robustness of the 10th percentile PNI value in comparison to optimal cut-off values for individual short- and long-term surgical outcomes.

Numerous prospective studies of perioperative nutritional support have failed to reveal improvements in short-term surgical outcomes as a result thereof.^29–31^ Thus, it is likely that only severely malnourished patients benefit from preoperative nutritional support.^31–33^ If malnutrition affects postoperative results and a clinically applicable parameter becomes available, interventions to improve nutritional status prior to surgery could become attractive targets to optimize patient outcomes. Since it is unknown whether PNI could serve as a nutritional parameter to select candidates for nutritional intervention, prospective validation of nutritional intervention in patients with low PNI should be performed in the future.

Regarding long-term surgical outcomes, our study showed that PNI was an independent risk factor for overall survival, but not for recurrence-free survival. Since our findings on recurrence-free survival do not corroborate those of a previous study,^21^ we extensively validated the prognostic significance of PNI. For further validation, we examined the performance of the 10th percentile cut-off value in comparison to optimal cut-off values derived from statistical tests. In doing so, we found that low PNI is indeed not a significant prognostic factor for recurrence-free survival in subgroup analysis stratifying patients by disease stage or in multivariate Cox analysis. Therein, the prognostic impact of PNI on recurrence-free survival decreased and disappeared after adjusting for confounding factors. Additionally, although PNI was significantly associated with overall survival, an age difference of 7 years (63.2 vs. 56.4) between the 2 groups may have affected the survival analysis results, despite adjusting for age in the statistical models. Contrary to previous reports, our findings showed PNI holds little prognostic value as a parameter for long-term surgical outcomes.

Despite extensive validation in a large cohort, retrospective inclusion and exclusion of patients, the collection of laboratory data, and the use of prospectively maintained databases, this study has inherent limitations related to its retrospective design. We also did not control for other variables affecting PNI. However, to the best of our knowledge, our study of the clinical significance of PNI in gastric cancer is the most comprehensive, to date and includes extensive comparison with statistically optimized cut-off values and adjustment for potential confounding factors.

In conclusion, PNI was not associated with cancer recurrence in the present study. Although low PNI patients showed unfavorable prognosis regarding overall survival, their advanced age may have affected the survival results, despite adjusting for age in multivariate analysis. The index, nevertheless, exhibits predictive capabilities for the stratification of patients at increased risk of postoperative morbidity and mortality. Moreover, this index may be of use in identifying candidate patients who would benefit from perioperative nutritional support to improve surgical outcomes.
